# Pan-Genome Reverse Vaccinology Approach for the Design of Multi-Epitope Vaccine Construct against *Escherichia albertii*

**DOI:** 10.3390/ijms222312814

**Published:** 2021-11-26

**Authors:** Khurshid Jalal, Kanwal Khan, Diyar Ahmad, Ajmal Hayat, Zarrin Basharat, Muhammad Naseer Abbas, Saad Alghamdi, Mazen Almehmadi, Muhammad Umar Khayam Sahibzada

**Affiliations:** 1 International Center for Chemical and Biological Science, H.E.J. Research Institute of Chemistry, University of Karachi, Karachi 75270, Pakistan; khurshid26523@gmail.com (K.J.); h.diyar438@gmail.com (D.A.); 2 Dr. Panjwani Center for Molecular Medicine and Drug Research, International Center for Chemical and Biological Science, University of Karachi, Karachi 75270, Pakistan; khankanwal011@gmail.com; 3 Department of Pharmacy, Abdul Wali Khan University, Mardan 23200, Pakistan; ajmalhayat953@yahoo.com; 4 Jamil-ur-Rahman Center for Genome Research, Dr. Panjwani Center for Molecular Medicine and Drug Research, International Center for Chemical and Biological Sciences, University of Karachi, Karachi 75270, Pakistan; 5 Department of Pharmacy, Kohat University of Science and Technology, Kohat 26000, Pakistan; dr_naseer86@yahoo.com; 6 Laboratory Medicine Department, Faculty of Applied Medical Sciences, Umm Al-Qura University, Makkah 21955, Saudi Arabia; ssalghamdi@uqu.edu.sa; 7 Department of Clinical Laboratory Sciences, College of Applied Medical Sciences, Taif University, Taif 21944, Saudi Arabia; Dr.mazen.ma@gmail.com; 8 Department of Pharmacy, Sarhad University of Science & Information Technology, Peshawar 25100, Pakistan

**Keywords:** pan-genome, reverse vaccinology, *Escherichia albertii*, chimeric vaccine, multi-epitope, immunoinformatics

## Abstract

*Escherichia albertii* is characterized as an emerging pathogen, causing enteric infections. It is responsible for high mortality rate, especially in children, elderly, and immunocompromised people. To the best of our knowledge, no vaccine exists to curb this pathogen. Therefore, in current study, we aimed to identify potential vaccine candidates and design chimeric vaccine models against *Escherichia albertii* from the analysis of publicly available data of 95 strains, using a reverse vaccinology approach. Outer-membrane proteins (n = 4) were identified from core genome as vaccine candidates. Eventually, outer membrane Fimbrial usher (FimD) protein was selected as a promiscuous vaccine candidate and utilized to construct a potential vaccine model. It resulted in three epitopes, leading to the design of twelve vaccine constructs. Amongst these, V6 construct was found to be highly immunogenic, non-toxic, non-allergenic, antigenic, and most stable. This was utilized for molecular docking and simulation studies against six HLA and two TLR complexes. This construct can therefore be used for pan-therapy against different strains of *E. albertii* and needs to be tested in vitro and in vivo.

## 1. Introduction

*Escherichia albertii* is an emerging gram-negative, mucocutaneous, non-motile, monophyletic bacterium, belonging to the *Enterobacteriaceae* family [1]. It is the causative agent of foodborne illness and diarrhea, mostly in young children [2]. For the first time, *E. albertii* was diagnosed in a 9-month-old diarrheic child in Bangladesh and identified through biochemical tests, as *Hafnia alvei* [3]. It was later named as *E. albertii* after M. John Albert, who described the species’ initial isolate [4]. Further analysis revealed that it belonged to the genus *Escherichia* and included virulence genes (eae and cdt). Hence, *E. albertii* is recognized as a close relative of *Escherichia coli* [5]. Similar to enteropathogenic and enterohemorrhagic *E. coli* (EPEC and EHEC), this enteropathogen has a type III secretion system (T3SS), which is encoded by the locus of enterocyte effacement. *E. albertii* has various unique or noteworthy genetic traits, including those responsible for known biochemical properties and virulence factors, as well as an active T3SS [6].

The emergence of antibiotic resistant strains of *E. albertii* has caused a seriously alarming situation, regarding enteric fever treatment around the globe. It necessitates therapeutic discovery for the treatment of its infections. Identification of novel vaccine targets is one of the best approaches in drug discovery pipeline. The availability of bacterial genome sequence data allows use of innovative processing methods, for the identification of bacterial therapeutic targets [7,8]. Traditional drug discovery and vaccine designing approaches are expensive and time-consuming. Hence, genome-based technology has emerged as a viable option for discovering novel therapeutic targets and promiscuous multi-epitopes vaccines against harmful pathogens [9]. Reverse vaccinology is a frequently used computational approach for the design of vaccines [10] It enables vaccine development and design based on information from an organism’s genome sequence, without the requirement to grow pathogens. Methodology involves constructing numerous fragments (epitopes) from the pathogen’s outer membrane proteins, in order to activate cellular and humoral immune responses, while reducing the adverse consequences [11].

In order to prioritize and build vaccine targets against various infectious pathogens, the reverse vaccinology approach has been extensively used, e.g., against Yellow fever [12], *Mycobacteroides abscessus* [13], *Acinetobacter baumannii* [14] infection. In the current study, we applied pan-genomic analysis to figure out the strain’s accessory, core, and unique genome. Based on conservation properties, core genes depicting non-homology to the human genes were employed to design a multi-epitope vaccine construct from the outer membrane protein of *E. albertii.* Since the reverse vaccinology technique uses a number of in silico filters to choose high-probability proteins as vaccine candidates from the whole coding DNA of the organism, we are certain that the findings of this study will help to speed up the development of vaccine against *E. albertii* by allowing for more experimental (in vitro and in vivo) testing of the modeled construct.

## 2. Results and Discussion

### 2.1. Core Genome and Vaccine Candidate Identification

Less than 2000 genes (n = 1863) were identified as core genome, shared by all strains of *E. albertii*. These genes were utilized for vaccine target mining. Core genome consisted of 0.86% (1863/216,586 = 0.8% CDS) of the accessory genome fraction.

The subtractive genomic approach was applied to find the essential protein coding genes, crucial for the survival of the pathogen. Essential genes are evolutionarily conserved, compared to the non-essential genes [15], classifying them as a potential therapeutic vaccine candidate. The advancement in informatics approaches makes it easier to identify such genes compared to conventional methods and databases have been compiled, based on this information. In the present study, we used two databases—(1) Database of Essential Genes (DEG) and (2) Cluster of Essential Genes (CEG)—for the identification of essential genes. The dataset of these databases was compared to the core genome and genes having sequence homology in both datasets were retrieved as essential genes. CEG identified 1058 genes while DEG identified 1135 genes as essential for *E. albertii*. Comparatively, 1041 genes were commonly identified from these two databases, which were selected for further analysis.

Furthermore, BLASTp was performed for essential genes against the whole proteome of human as well as gut proteome to identify non-homologous vaccine candidates, using a cut-off value of 0.0001 (E-value 10^−3^). The result identified 532 proteins as non-homologous to human proteome and 64 proteins as non-homologous to the gut bacteria. Among these 64 proteins, only 4 proteins were outer membrane proteins, as predicted by PSORTb. Identification of the sub-cellular localization is one of the crucial steps to reduce time, labor, and resources for the identification of the best vaccine targets and therapeutic agent design. The identified outer-membrane proteins were used further to construct a multi-epitope vaccine.

### 2.2. Antigenicity Prediction

Antigens are the molecules exposed to the host by the pathogen, which induce host immune response. The antigenicity analysis for shortlisted outer-membrane proteins through VaxiJen v2.0 server was found to be 0.7 for outer membrane Fimbrial usher (FimD), 0.57 for fimbrial outer membrane usher protein, 0.66 for Porin OmpC protein, and 0.6 for Porin OmpF protein respectively. A cut-off value of 0.5 was used. Based on the antigenicity scores, Fimbrial usher (FimD) outer membrane protein was shortlisted as a vaccine candidate.

### 2.3. MHC-I Epitope Mining

In order to attain the T-cell epitopes, the sequence of shortlisted outer-membrane protein was fed to NetCTL server. It resulted in the identification of 870 epitopes from FimD protein. These identified epitopes were further subjected to The IEBD server, resulting in the generation of 891 MHC-1 epitopes. By applying the cut-off score of ≥ 0.2 to 0.04, percentile rank based prediction for these epitopes led to the identification of only 14 MHC-I epitopes. The redundant MHC-I epitopes were removed, resulting in the final selection of only 8 MHC-I epitopes.

The epitopes of the MHC Class-I molecules were identified to detect distortion, such as an infection. Several studies reported that immunogenicity of the peptide is dependent upon the amino acid sequence. Higher number of aromatic amino acids present in the peptides are more immunogenic than other peptides. The proficiency of epitopes to induce T-cell response is based on the level of immunogenicity score. Therefore, the 8 shortlisted MHC-I epitopes were examined for immunogenicity prediction, using a cut-off value of the positive predicted scores. The IEBD immunogenicity analysis revealed 6 (out of 8) epitopes as most immunogenic epitopes.

Additionally, for the evaluation of toxicity level, online tool ToxinPred was used. It predicted that all 6 epitopes were non-toxic (do not cause any harm) to the host cell. These non-toxic epitopes were then subjected to VaxiJen tool for the analysis of antigenicity with a cut-off value of 0.5. The VaxiJen result showed that out of 6 epitopes, 4 epitopes were more antigenic and were selected for further evaluation as shown in Table 1.

### 2.4. MHC-II Epitope Prediction

Additionally, the FimD protein was also used to identify MHC-II epitopes, using the IEDB server. The epitopes having binding affinity <200 nM and percentile ranks <0.2 were shortlisted and used for further analysis. The results showed that total 18,036 epitopes were generated, while only 7 were shortlisted by applying the cut-off value of <0.2 percentile rank (Table 2).

### 2.5. MHC Restricted Alleles Cluster Analysis

**Figure 1:**
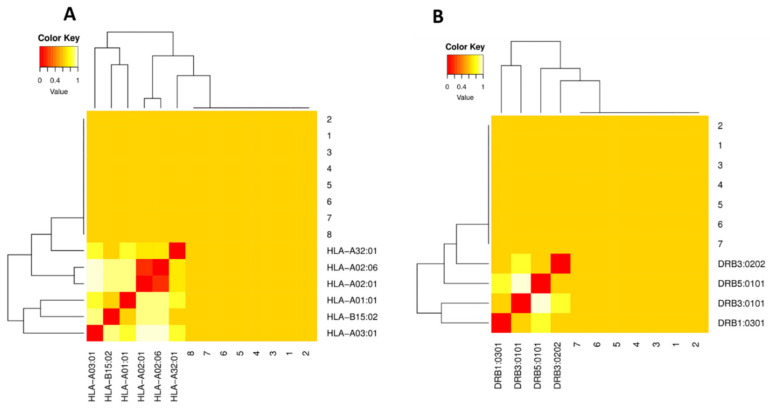
Clustering analysis for MHC class I and II epitopes. (**A**) The cluster analysis of MHC molecules and HLA alleles (MHC class I clustering alleles). (**B**) MHC class II clustering alleles. Red color indicates strong interaction while yellow zone indicates a weaker interaction.

Clusters of MHC restricted alleles and their appropriate peptides were re-evaluated by cluster analysis. It resulted in the construction of heat map of MHC-I and MHC-II, respectively. Epitopes clustered are formed on the basis of their interactions with the human leukocyte antigen (HLA). The yellow color represents weaker interactions while red color shows strong interactions, with proper annotation (Figure 1).

### 2.6. B-Cell Epitope Prediction

Apart from cellular immunity (MHC-I/II epitope prediction), B-cell epitopes were also predicted using different online tools, to assess potential induction of humoral immunity. In order to eliminate the pathogen, humoral immunity is also necessary, besides cellular immunity. Hohman and Peters suggested that vaccines are generally thought to work by generating memory B cells that, upon exposure to infectious challenge, rapidly produce antibodies (Abs) which mediate pathogen clearance by phagocyte or complement-mediated pathways [16]. The Bacillus Calmette–Guérin (BCG) vaccine against tuberculosis is the only licensed vaccine believed to work primarily through cell-mediated immunity [17]. The prediction and classification of B-cell epitopes play a vital role in vaccine designing, immunodiagnostic tests, and antibody production. For our study, the BCPred server generated 19, FCPred 32, while ABCPred generated 89 B-cell epitopes respectively (Supplementary Table S1).

**Figure 2:**
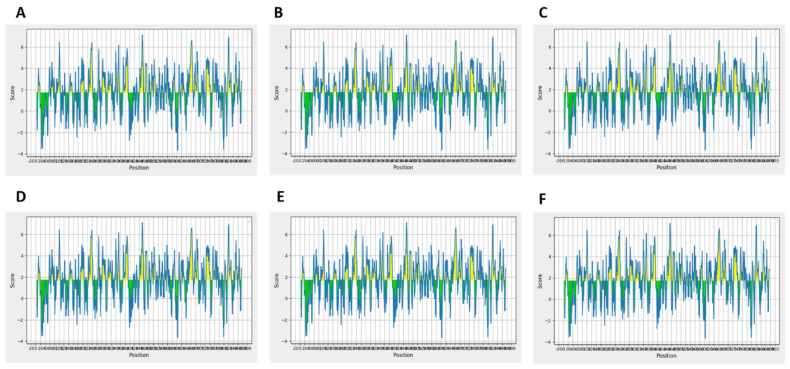
B-cell epitope analysis. (**A**) Bepipred linear epitope. (**B**) Chou and Fasman beta-turn prediction. (**C**) Emini surface accessibility prediction. (**D**) Karplus and Schulz flexibility prediction. (**E**) Kolaskar and Tongaonkar antigenicity. (**F**) Parker hydrophilicity prediction.

Moreover, resultant B-cells epitopes were further examined and shortlisted on the basis of BepiPred linear epitope prediction (Figure 2A), Chou–Fasman beta-turn prediction (Figure 2B), Kolaskar Tongaonkar antigenicity (Figure 2C), Emini surface accessibility (Figure 2D), Karplus–Schulz flexibility (Figure 2E), and Parker hydrophilicity (Figure 2F) prediction parameters. Furthermore, we compared all the epitopes generated by BCpred, FBCpred, and ABCpred in order to finalize the similar epitopes predicted through all these tools. The result revealed that 26 epitopes (Supplementary Table S2) were similar among all these predicted epitopes and were used for further analysis.

### 2.7. Predicted Epitope Comparison for Vaccine Construct

The predicted B-cell, MHC-I, and MHC-II epitopes were manually compared with each other to finalize the similar epitopes present in the B-cell, MHC-I, and MHC-II epitopes for the making of final vaccine construct. These are considered as having capability to stimulate B-cell, MHC-II, and MHC-II molecules. Finally, we shortlisted only 3 similar epitopes based on similarities among the B-cell and MHC-I and MHC-II epitopes i.e., LNLSVYQRNTQCLHNRKLRLAGFFVRLSVA, TAGEYRSGNAQQEKPRFFQSTLLHGLPAGWTIYGGMQLADRYR, and LSNFENGQELPPGTYRVDIYLNNGYMATRDVTFNAGDSE, respectively (Supplementary Table S3).

Consequently, the shortlisted B-cell, MHC-I, and MHC-II epitopes were linked sequentially with corresponding adjuvant, PADRE sequence, GGGS, and EAAAK linker to design the different combinations of vaccine constructs. Various combinations of epitope sequences were linked with four different adjuvants. Among these, beta-defensin is an antimicrobial peptide that has a vital role in innate immune response. It may also contribute in the immune response induction by recruiting dendritic cells (DCs), monocytes, and T cells to site of inflammation [18]. The innate immune system protects the host from microbial diseases such as bacteria, viruses, parasites, and fungi as a first line of defense. It is made up of cells and molecules that are designed to recognize and respond to a variety of microbial stimuli. A set of germline-encoded receptors and secreted proteins have been developed to identify pathogen-associated molecular patterns, which are frequent, conserved, and essential microbial characteristics (PAMPs) [19,20]. Other adjuvant was L7/L12 ribosomal protein, which is involved in the start, elongation, and termination of translation by the 70 S ribosome. The presence of L7/L12, which is required for ribosomal translocation, is required for EF-GTPase G’s activity [21], The *heparin-binding haemagglutinin (HBHA)* protein adjuvant is an immunodominant antigen that stimulates T cells and causes them to produce interferon-gamma (IFN-gamma) [22], and HBHA conserved sequence, respectively [23]. Apart from these, the use of linkers boosts the immunogenicity whereas PADRE sequence helps in the initiation of CD4+ cells [24]. Twelve vaccines constructs were made with different combinations of adjuvants and linkers, as shown in the Supplementary Table S4.

### 2.8. Antigenicity, Allergenicity, Solubility, and Physiochemical Properties Analysis

The antigenicity, allergenicity, solubility, and physiochemical properties of these twelve vaccine constructs were assessed. The construct with AlgPred score predicted higher than -0.8 was considered as allergenic vaccine. The result showed that out of twelve constructs, five were allergenic. These were, therefore, excluded. Remaining seven constructs (V2, V3, V4, V6, V8, V10, V11) were assessed for their solubility and antigenicity. All seven constructs showed a high level of solubility and antigenicity scores i.e., >0.8, predicted with a default threshold of 0.5.

The physicochemical properties (i.e., hydropathicity index, number of amino acids, aliphatic index, PI value, molecular weight, and instability index) of all seven shortlisted vaccine constructs were assessed through ProtParam server. The molecular weight was estimated to be 24–46 kDa with a pI score of 5.6–9.2, whereas the instability index (II) value was found to be stable for all shortlisted vaccine constructs i.e., between 24–36. The grand average of hydropathicity was found to range between −0.3 and 0.4, enough to initiate an immunogenic reaction response (Table 3).

### 2.9. Vaccine Construct Structure Prediction and Validation

**Figure 3:**
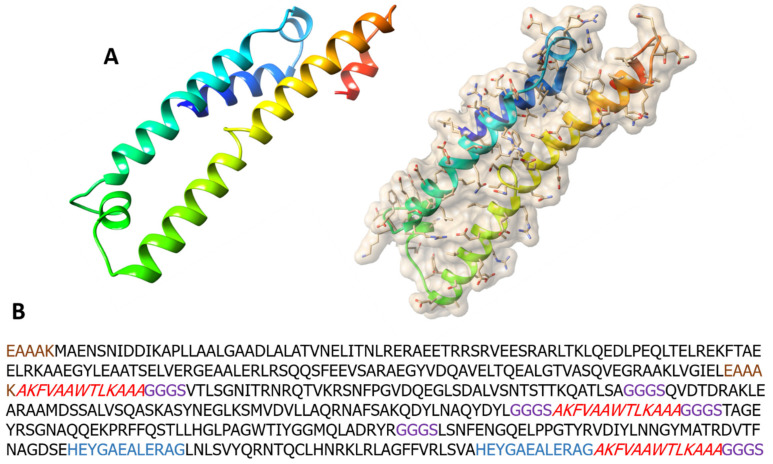
Vaccine structure modeling and validation. (**A**) The 3D model of a multi-epitope vaccine construct, obtained by Swiss Model. (**B**) Vaccine construct sequence with adjuvants (EAAAK-EAAAK (shown in brown color), G-linker (shown in purple), H-linker (shown in blue), and PADRE Sequences (shown in red).

The 3D structure of seven constructs was modeled through Swiss Model tool [25]. On the basis of modeled structure and template sequence similarities, V6 vaccine construct was finalized as ultimate construct. Selection of model was purely based on the presence of a high percentage of residues in the most favorable region of the Ramachandran plot. The template of this V6 construct was apolipoprotein E of humans, with PDB ID: 6NCN (Figure 3). In terms of stereochemical quality, the modeled structure showed that 91.1% residues lie in the most favorable region, and 8.1% residues in additionally allowed region (Supplementary Figure S1A). PSIPRED tool was used to predict and validate the 2D molded structure of vaccine. The structure of vaccine construct showed a similar number of alpha helices and beta turns, as predicted by Swiss Model (Supplementary Figure S1B).

### 2.10. Molecular Docking Studies for V6

**Figure 4:**
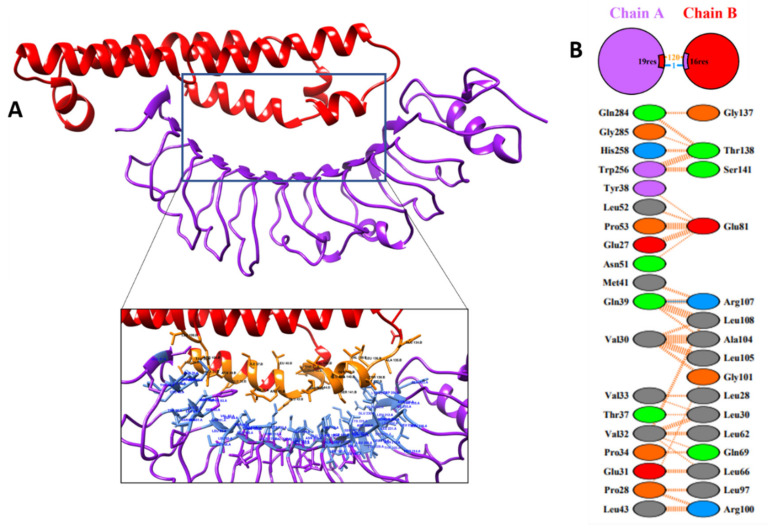
Docked vaccine construct with TLR4/MD. (**A**) Docked complex of vaccine construct (red) and TL4/MD (purple). (**B**) Non-bonded interactions between the vaccine model and TLR4/MD protein shown in orange. Blue line represents hydrogen bond between the docked complex.

The TLRs are involved in innate immune responses that can potentiate adaptive immune responses. A potent multi-epitope vaccine construct has the ability to boost the immune response against a number of epitopes via binding TLR molecules and HLA alleles. The shortlisted V6 vaccine construct was docked with the six different HLA alleles: (1) protein with PDB ID: 3C5J (HLA-DR B3^∗^02:02), (2) protein with PDB ID: 1H15 (HLA-DR B5^∗^01:01), (3) protein with PDB ID: 2FSE (HLA-DRB1^∗^01:01), (4) protein with PDB ID: 2Q6W (HLA-DR B3^∗^01:01), (5) protein with PDB ID: 2SEB (HLA-DRB1^∗^*04:01), and (6) protein with PDB ID: 1A6A (HLA-DR B1*^∗^03:01) and TLR protein, using PatchDock. Obtained models were refined through FireDock server. The PatchDock docking results with -13.96 binding energy suggested a good interaction between V6 and TLR-4/MD2 complex (Table 4). It shows one hydrogen bond, 120 non-bonded interactions while no salt bridge was observed. The protein–protein interaction of V6 construct and TLR4/MD showed that Arg107-Gln39 amino acids make contact along with other interactions, as highlighted in Figure 4.

### 2.11. Molecular Dynamics and Immune Simulation Studies for Construct V6

**Figure 5:**
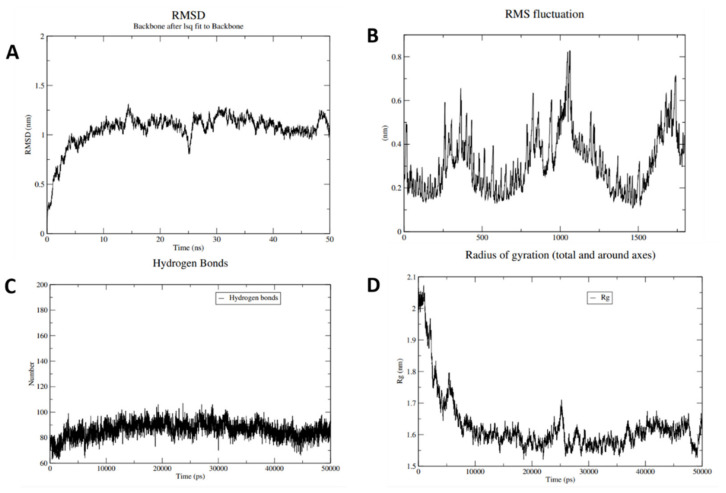
Molecular dynamic simulation of construct V6. (**A**) Root mean square deviation (RMSD) of protein backbone. (**B**) Root mean square fluctuation of vaccine molecule. (**C**) Hydrogen bonds of vaccine construct. (**D**) Plot of radius of gyration for V6.

The molecular dynamics simulation was performed for the best docked model to validate the complex interactions and flexibility. GROMACS was used to find the movement of molecules and atoms of vaccine construct, for 50 ns. It was observed that the complex was found to be stable after 30 ns with mild fluctuations (Figure 5). Furthermore, iMODs simulation analysis revealed deformability graph for the stability and mobility of vaccine-protein complex. It highlights the region of protein having deformability, illustrated in terms of the peaks. The eigenvalue of the protein and vaccine complex was found to be 1.42∗10^−4^, while the variance association plot representing the cumulative variance of complex was also obtained. Individual variance is depicted by red color. B-factor graph results aid in the clear visualization of the docked complex as shown in Supplementary Figure S2.

**Figure 6:**
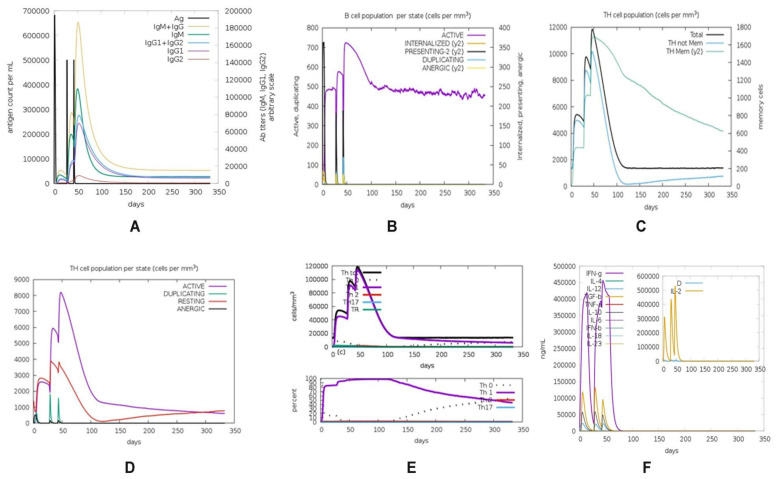
C-ImmSim presentation of an in silico immune simulation with the construct. (**A**) Immunoglobulin production in response to antigen injections (black vertical lines); specific subclasses are shown as colored peaks and (**B**) The evolution of B-cell populations after the three injections. (**C**) T-helper cell populations per state after the injections. (**D**) The evolution of T-cytotoxic cells. (**E**) Production of natural killer cells. The resting state represents cells not presented with the antigen while the anergic state characterizes tolerance of the T-cells to the antigen due to repeated exposures. (**F**) The main plot shows cytokine levels after the injections. The insert plot shows IL-2 level with the Simpson index. D is shown by the dotted line. D is a measure of diversity. Increase in D over time indicates emergence of different epitope-specific dominant clones of T-cells. The smaller the D value, the lower the diversity.

The final selected vaccine construct was used to perform a simulation of vaccine construct under different conditions to analyze the human immune system response with C-ImmSim software. The ImmSim server immune simulation outcomes confirmed consistency with real immune reactions. The C-ImmSim server resulted in the prediction of B-cell, T-Helper, T-cytotoxic, natural killer cells, interleukins, and Ab production. The primary response was illustrated by high IgM levels. In addition, decrease in antigenic concentration was observed, with an increase in the immunoglobulin expression i.e., B-cell population, IgG1+IgG2, IgM, and IgG+IgM. The results showed a clear increase in the population of Th (helper) and Tc (cytotoxic) cells with memory growth after the induction of V6 construct. The IFN-g production was also identified and has been stimulated after immunization, as shown in Figure 6.

### 2.12. Codon Optimization and In Silico Cloning

**Figure 7:**
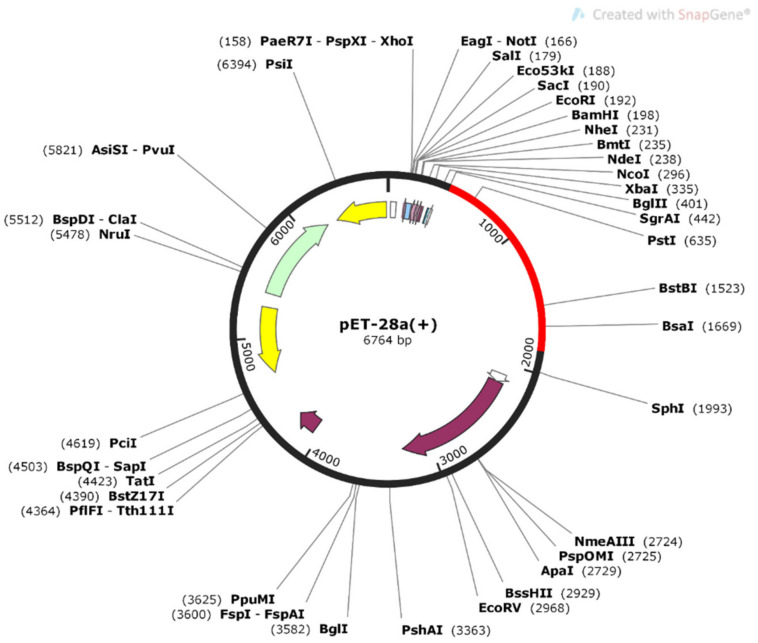
Codon optimization and in silico cloning of vaccine model. In silico restriction cloning of the multi-epitope vaccine sequence into the pET30a (+) expression vector using SnapGene software. The red part represents the vaccine’s gene coding region, and the black circle represents the vector backbone.

The JCAT tool was used for the codon optimization and cloning of V6. Construct V6 was reverse translated for best expression in *E. coli* (strain K12). The average GC content and Codon Optimization Index (CAI) value for V6 was predicted to be 53.2% and 0.94 respectively, resulting in the successful expression of vaccine construct in *E. coli* system. Finally, SnapGene tool was used to introduce the adapted codon sequence (V6) to construct the recombinant plasmid, into the pET30a (+) vector (Figure 7).

## 3. Discussion

New vaccines are needed to combat the rising issue of diseases and emergence of resistant microbes. In this study, we worked on constructing one against *E*. *albertii,* which is one of the notorious pathogens responsible for food-borne infections. It is identified as facultative anaerobic, monophyletic, non-motile, and Gram-negative bacteria, considerably linked to diarrheal illness in children [26]. It is an emerging pathogen of importance that requires a therapeutic measure for prevention and cure. Computation based analysis utilizes software programs and databases for designing of multi-epitope vaccine, reducing the conventional laboratory-based experimental practice.

Herein, we applied the subtractive pan-genome analysis, followed by a reverse vaccinology approach on 95 strains, to identify vaccine candidates and design a novel vaccine construct against *E. albertii*. Through pan-genome analysis, we identified only 4 outer membrane proteins, i.e., FimD, fimbrial outer membrane usher protein, Porin OmpC, and Porin OmpF. The outer membrane proteins play important roles in bacterial pathogenesis [27] such as invasion, adhesion, effector secretion, biofilm formation, and cell-to-cell dissemination [28]. Furthermore, the antigenicity analysis showed that FimD is significantly antigenic, having a score of 0.7, therefore, it was selected for further studies. Moreover, FimD protein has been identified as a potent vaccine candidate against *A. baumannii* [29] and *Gallibacterium anatis* [30]. The immunogenic MHC-I (n = 8), MHC-II (n = 7), and B-cell epitopes (n = 3) were identified from FimD protein. Twelve different combinations of vaccines were constructed from these shortlisted common epitopes, using PADRE sequences, E-linker EAAAK, G-linker GGGS, and H-linker, along with four different adjuvants i.e., HBHA protein, HBHA conserved sequence, beta-defensin, and L7/L12 ribosomal protein, respectively. The twelve constructs were further extensively analyzed for toxicity, immunogenicity, conservancy, pattern of allergenicity, physio-chemical properties, structural stability, and structure stereochemistry. Based on these criteria, only V6 construct was found to be the most favorable vaccine construct. The structure of V6 was modeled using Swiss Model and validated through Procheck and PsiPred. The interactions of modeled vaccine construct with Human Leukocyte Antigen (HLA) and TLR4 to elucidate effective immune response were studied using molecular docking simulation. The TLR4 and V6 complex resulted in the binding energy of −8.9, mediating one hydrogen bond with Arg107-Gln39 and 120 non-bonded interactions. Additionally, the vaccine model was simulated under the in vivo conditions, to check its stability using GROMACS. The molecular dynamics simulation of the vaccine for 50 ns displayed the stability of vaccine model at 30ns. Furthermore, V6 showed the potential to elicit a significant immunological response, according to immune simulation studies. A high cytokine response and a large number of B memory cells may help clear infections and avoid reinfection. The codon optimization of V6 model was followed by reverse translation to its cDNA to ensure a successful expression in *E. coli* pET-28a(+) expression vector. The GC and CAI values predicted for V6 were 53% and 0.94 respectively, depicting successful expression of vaccine.

Our current findings suggest a set of novel proteins that might be exploited as vaccine candidates in combination with a chimeric vaccination model against *E. albertii*. The methods used in this study are an appealing alternative way to combating the spread of *E. albertii* resistant strains. This research can serve as a standard for future experimental and clinical testing of vaccination models in animal models for their function in protecting the host against *E. albertii* pathogenicity.

## 4. Material and Methods

In the current study, a pan-genomic analysis based reverse vaccinology approach was utilized to assess the novel potential vaccine candidate and design multi-epitope vaccine construct against *E. albertii*. The detailed steps are mentioned below:

### 4.1. Pan-Genomics and Vaccine Target Prediction

Entire genome of *E. albertii* strains (n = 95) was retrieved from the NCBI database and subjected to pan-genome analysis, employing BPGA software according to Basharat et al. [31,32]. Core genome was retained for vaccine target mining.

Using subtractive genomic technique, core genome was exposed to pharmacological vaccine target mining. First of all, the CD-HIT [33] was used to eliminate paralogous sequences from the core genome sequences, using 60% cut-off value for the sequence similarity. Essential genes with an E-value of 10^−10^ and a bit score of 100 were utilized to identify essential genes from both the CEG [34] and DEG [35] databases.

Coding DNA sequences were translated and using BLASTp, non-homologous sequences to the human host (with an E-value > 0.005) and intestinal flora (E-value > 10^−4^) were filtered out. Furthermore, vaccine candidates were predicted with an E-value < 10^−3^. A gap extension penalty of 1 and gap penalty of 11 were used as standard. Differential analysis was carried out on 83 distinct species of human microbial gut flora in order to assess the uniqueness of our targets, which did not show any sequence similarities to typical gut flora [36]. To distinguish non-homologous proteins, an E-value cut-off of 10^−2^ was chosen based on an extensive literature survey [37]. The major aim of this evaluation was to prevent adverse side effects against human and essential or beneficial microbial gut flora. Only non-homologous proteins to the human host and gut flora were chosen for further study and further assessed by PSORTb v.3.0 [38], for subcellular localization prediction. It classified proteins as cytoplasmic, outer-membrane, extracellular, and cell wall proteins.

### 4.2. Immunoinformatic Analysis

The predicted outer membrane proteins, identified through PSORTb were selected as vaccine candidates for chimeric or multi-subunit vaccine construction. Vaxijen v2.0 [39] was used with a threshold value of 0.5 to examine these proteins for antigenicity evaluation and highly antigenic protein was selected for vaccine designing.

### 4.3. MHC-I T-Cell Epitope Prediction

The NetCTL server [40] was used to find T-cell epitopes that might activate the human immune system and create memory cells (immunomodulatory effects). The predicted epitopes were selected on the basis of these factors: (1) overall intrinsic peptide potential scores combined with transporter associated efficiency prediction, (2) protease cleavage, (3) prediction score for MHC I epitope affinity, (4) a collective score of predicted parameters with a threshold value of 0.75.

The binding analysis of predicted T-cell epitopes was further investigated using the Immune Epitope Database and Analysis Resource (IEDB AR) server [41], where T-cells recognize antigen represented by MHC-I. Standard parameters from consensus of these methods, i.e., NetMHCpan [42], CombLib [43], SMM [44], and ANN [45] were obtained, whereas, for MHC-I prediction, all HLA alleles were utilized. The HLA alleles chosen for the MHC-I investigation were HLA-A2, HLA-A 2.1, HLA-A3, HLA-B 5401, HLA-A 0205, HLA_0201, and HLA-B 5102. The threshold parameters based on IC50 <100 nM and percentile rank (<0.2) were considered as cut-off values for the shortlisting of MHC-I epitopes [46].

Notably, the anticipated MHC-I epitopes should have adequate immunogenicity to activate CD4 or CD8 T lymphocytes. Consequently, the IEBD AR [47] tool was utilized to predict MHC-I immunogenicity. The positive score value for MHC-I epitopes was chosen for further investigation. Additionally, the toxicity, conservancy, and antigenic characteristics of the MHC-I epitopes that were shortlisted and had a high immunogenic score were further scrutinized by ToxinPred server [48] with a cutoff value of 0.5, with an accuracy of 70–80% at IEBD server [49], and probability threshold score of 0.5, respectively, at VaxiJen server [39].

### 4.4. T-cell MHC-II Prediction and Cluster Analysis

IEBD server was used to identify MHC-II epitopes using a consensus method. The cut-off value for shortlisting MHC-II epitopes was set at <0.2 peptide rank and IC_50_ < 100 nM for top binders against the 95% HLA variability found in human population worldwide i.e., DRB1^∗^0101, DRB1^∗^1301, DRB1^∗^0301, DRB1^∗^0401, DRB1^∗^0701, DRB1^∗^0801, DRB1^∗^1101, DRB3^∗^01:01, HLA- HLA-DRB3^∗^02:02, HLA-DRB4^∗^01:01, HLA-DRB5^∗^01:01, and DRB1^∗^1501 [46]. In the current study, multiple immunogenic epitopes with a length of 9–14 residues were selected for further analysis.

For further confirmation of shortlisted MHC-I/II epitopes, the MHCcluster server [50] was utilized to cluster MHC restricted alleles with appropriate MHC epitopes. This tool produces a heat map and phylogenetic tree illustrating the functional connection between HLAs and epitopes, as well as clustering of MHC-I and II epitopes.

### 4.5. B-Cell Epitope Prediction

An ideal peptide vaccine should be capable of eliciting long-lasting humoral immunity, similar to that elicited by some infections. The objective of B-cell epitope prediction was to ascertain the antigen recognition by B lymphocytes that can trigger humoral immunity. B-cell epitopes stimulate humoral immunity, which have the potential to eradicate pathogens by producing antibodies against antigens exposed in the human body. Vaccines are hypothesized to operate by creating memory B cells, which create antibodies (Abs) that drive pathogen clearance via phagocyte or complement-mediated pathways when exposed to an infectious challenge [16]. The only approved TB vaccine, the bacillus Calmette–Guérin (BCG) vaccine, is thought to act largely through cell-mediated protection [17]. B-cell epitopes were identified employing ABCpred, FBCpred, and BCpred [51] servers that apply sequence-based methods with cut-off scores of >0.51 and 75% specificity. In addition, the ElliPro server [52] was utilized to classify B-cell epitopes based on their hydrophobicity content [53], antigenicity [54], flexibility [55], accessibility, beta-turn prediction through Chou and Fashman tool [56].

### 4.6. Epitope Selection and Designing Vaccine Construct

Epitopes that may activate immune cells (B and T cells) are important for the development of epitope-based vaccines [46]. Therefore, binding affinity and similarity were determined among MHC I/II and B-cell epitopes of *E. albertii* outer membrane protein. The manual comparison of identified MHC-I, MHC-II, and B-cell epitopes was carried out and the overlapped epitopes were selected for making vaccine constructs.

We looked at several combinations of sequence assemblies to create a new vaccine with low toxicity, allergenicity, and high immunogenicity. For this purpose, shortlisted epitopes were sequentially conjugated with appropriate adjuvants (beta-defensin, HBHA protein, HBHA conserved sequence, and L7/L12 ribosomal protein, PADRE (Pan HLA-DR reactive epitope), and linkers (GGGS, HEYGAEALERAG, and EAAAK) [46]. The PADRE peptide activated CD4^+^ T-cells, which improved the peptide vaccine’s effectiveness and potency. Adjuvant HBHA and L7/L12 ribosomal protein are agonists of TLR4/MD2 complex whereas beta-defensin adjuvant is an agonist to TLR1, TLR2, and TLR4. HTL, CTL, and B-cell epitopes were conjugated using HEYGAEALERAG and GGGS linkers, whereas adjuvant sequences at both the N and C-terminus were joined using EAAAK linkers [46]. The design vaccine constructs were then further analyzed.

### 4.7. Assessment of Vaccine Constructs and Structure Modeling

Adverse allergic reactions may be linked with vaccine outcomes. In order to evaluate the allergic features of the built vaccine model, AlgPred tool [57] was utilized with a cut-off score of -0.4 and 85% accuracy to inspect the allergenicity. Antigenic nature of vaccine models was predicted by using VaxiJen and ANTIGENpro server [58] with a threshold value of >0.5. Moreover, SOLpro program was used with 74% accuracy and corresponding probability (≥0.5) for the prediction of vaccine solubility, upon expression in *E. coli* [58].

The Expasy ProtParam tool [59] was used to perform physicochemical and functional evaluation of vaccines based on pK values of various amino acids, GRAVY values, instability index, estimated half-life, hydropathicity, molecular weight, aliphatic index, and isoelectric pH parameters [59]. It is important to assess physicochemical properties to ascertain the safety and efficacy of vaccine candidates.

Swiss model server was used to model 3D structure of vaccine construct, whereas, psipred [60] and procheck [61] were applied for the validation of secondary and tertiary structure respectively. For additional structure-based investigation, the best-modeled vaccine design was chosen.

### 4.8. Molecular Docking Studies

PatchDock, a bioinformatics tool [62], was used to find interactions between the final modeled vaccine and six distinct human leukocyte antigen (HLA) alleles, i.e., 1H15 (HLA-DR B5^∗^01:01), 2FSE (HLA-DR B1^∗^01:01), 1A6A (HLA-DR B1^∗^03:01), 2SEB (HLA-DRB1^∗^04:01), 3C5J (HLA-DRB3^∗^02:02), and 2Q6W (HLA-DR B3^∗^01:01), were downloaded from the protein databank (PDB) (https://www.rcsb.org; accessed on 15 August 2021) [63]. The PatchDock docked complexes were refined and re-scored using the FireDock (fast interaction refinement in molecular docking) service, which gave the top 10 best solutions for final refinements based on global binding energy and binding score. [64]. Additionally, GRAMMX tool [65] was used to validate the docking step for the vaccine and TLR4/MD complex based on accuracy, interactions similarity, and hydrogen bonding pattern. The best model of vaccine complex visualization and interactions was interpreted using UCSF chimera [66] and PDBsum [67], respectively.

### 4.9. Molecular Dynamics and Immuno Simulation Studies

GROMACS (GROningen MAchine for Chemical Simulations) [68] was used to execute molecular dynamics simulation (MDS) and energy minimization to assess the vaccine construct’s stability and flexibility. This helped infer how the vaccine model behaves in a biological system. Topology files required for energy minimization and equilibrium were created and the solvation was executed with SPC216 water model, with steepest energy minimization algorithm while NVT and NPT were chosen ensembles, for 50,000 steps (100 ps) at 1 atm pressure and 300 K temperature. In addition, charged ions were added to neutralize the vaccine construct in the MDS system. Eventually, the vaccine MDS was carried out for 50ns to determine RMSD, root mean square fluctuation (RMSF), radius of gyration (Rg), and hydrogen bonds. MDS of docked complex (vaccine with TLR4) was carried out using the iMODs server [69], which is a rapid and free to use normal mode analysis based server. It can be used for defining and quantifying protein flexibility and stability in terms of B-factors, eigenvalue, covariance, and deformability.

Using the tool C-ImmSim [70], we were able to determine the immunogenicity and immune response profile of a chimeric peptide vaccine. The vaccine was administered at three different intervals for four weeks while the simulation was kept at its default settings with time periods of 1, 82, and 126 as reported by Rahman et al. [9] (8 h corresponds to one cell division cycle in real life), and random seed at 12345, with vaccine injection containing no LPS (lipopolysaccharide). The volume and steps of the immuno-simulation were adjusted to 10 and 1000, respectively, with homozygous host haplotypes HLA-DRB1^∗^0101, and HLA-DRB1^∗^0401, HLA-A^∗^0101, HLA-A^∗^0201, HLA-B^∗^0702 [9].

### 4.10. In Silico Cloning and Codon Optimization of Final Vaccine Construct

The Java Codon Adaptation Tool (JCAT) [71] was utilized to reverse translate the vaccine amino acid sequence to cDNA, for designing and expressing vaccine construct in *E. coli* vector, using a codon adaptation method. The JCAT tool was used to calculate the GC content of DNA sequences as well as the codon adaption index score (CAI) for the optimal nucleotide sequence while eliminating prokaryotic ribosome binding sites and termination of Rho-independent transcription cleavage sites for restriction enzymes [72]. Finally, the adapted codon sequence was inserted into the pET-28a (+) vector using the SnapGene (available at https://www.snapgene.com/; accessed on 17 August 2021) cloning module.

## 5. Conclusions

The current study applies the integrated immunoinformatic analysis of B-cell, and T-cell epitopes, based on subtractive genomics and reverse vaccinology approach to design the chimeric vaccine against *E. albertii.* The designing of an effective final vaccine construct (V6), through predicted epitopes was possibly made through the addition of appropriate linkers and adjuvant, that could elicit immune response. V6 was shortlisted as a potent vaccine candidate against *E. albertii* after the allergenicity, antigenicity, solubility, physiochemical analysis criteria were met. Additionally, the stability of V6 was also identified through MDS. Immune simulation also confirmed the infliction of immune response after the injection of V6. It also showed significant expression in *E. coli* vector pET30a (+) plasmid, back-translated to cDNA. However, further in vitro, animal studies and pre-clinical analysis are suggested to be performed for the validation of our predicted vaccine model as either recombinant or DNA vaccine, for the management of *E. albertii* infection.

## Figures and Tables

**Table 1:** MHC-I finalized epitopes along with predicted immunogenicity, antigenicity, and toxicity.

S. No	MHC−I Epitopes	Immunogenicity	Antigenicity	Toxicity
1	YTANAAEIY	0.24338	0.5146	Non—Toxic
2	GTANAAEIY	0.24338	0.8780	Non—Toxic
3	LTGYGQWEY	0.17668	−0.4627	—(excluded based on antigenicty)
4	YQRNTQCLH	0.17447	0.5174	Non—Toxic
5	GSIDYGRNY	0.13372	−0.3789	−
6	FADVGSIDY	0.05129	0.0453	Non—Toxic
7	QINSDLTGY	−0.10485	−	
8	YFNKNMSTY	−0.50764	−	−

**Table 2:** MHC-II epitopes.

S. No.	MHC−II Epitopes	Antigenicty	Toxicity
1	KPRFFQSTLLHGLPA	0.2334	Non—Toxic
2	EKPRFFQSTLLHGLP	−0.1383	Non—Toxic
3	YRVDIYLNNGYMATR	0.7947	Non—Toxic
4	RVDIYLNNGYMATRD	0.7810	Non—Toxic
5	QEKPRFFQSTLLHGL	−0.1147	Non—Toxic
6	NRKLRLAGFFVRLSV	0.5390	Non—Toxic
7	RKLRLAGFFVRLSVA	0.4578	Non—Toxic

**Table 3:** Allergenicity, antigenicity, solubility predicted for 12 vaccine constructs.

Vaccine	Allergenicity	Antigenicity	Solubility	Molecular Weight	Theoretical pI	Instability Index	Aliphatic Index	GRAVY
**V1**	−1.02	0.77	0.90	-	-	-	-	-
**V2**	−0.69	0.89	0.95	49,983.58	5.61	34.66	79.06	−0.383
**V3**	−0.87	0.80	0.97	46,955.20	5.40	36.85	79.98	−0.397
**V4**	−0.60	0.80	0.94	46,955.20	5.40	36.85	79.98	−0.397
**V5**	−1.10	0.76	0.91	-	-	-	-	-
**V6**	−0.62	0.88	0.96	46,955.20	5.40	36.85	79.98	−0.397
**V7**	0.02	0.79	0.98	-	-	-	-	-
**V8**	0.23	0.82	0.95	46,955.20	5.40	36.85	79.98	−0.397
**V9**	−0.69	0.79	0.91	-	-	-	-	-
**V10**	−0.61	0.89	0.95	49,588.20	5.53	34.40	79.91	−0.376
**V11**	−0.62	0.85	0.98	24,674.97	9.62	24.82	68.68	−0.423
**V12**	0.23	0.79	0.94	-	-	-	-	-

**Table 4:** Docking result of vaccine 6 construct and human leukocyte antigen.

Receptor	Ligand	Solution Number	Global Energy	Attractive VdW	Repulsive VdW	ACE	HB
**1A6A**	V6	2	−9.41	−44.31	29.91	14.23	−7.40
**1H15**		10	5.19	−1.83	0.00	0.91	0.00
**2FSE**		4	−12.31	−47.21	46.21	15.02	−3.49
**2Q6W**		3	6.12	−0.97	0.00	−0.72	0.00
**2SEB**		5	−15.95	−23.87	5.53	8.00	−3.15
**2Z65**		3	−13.96	−24.00	25.01	20.87	−3.83
**3C5J**		2	17.19	−14.67	0.14	10.92	0.00

## Data Availability

All data generated or analyzed during this study are included in this published article (and its supplementary information files).

## Supplementary Materials

The following are available online at https://www.mdpi.com/article/10.3390/ijms222312814/s1.
