# Supplementary material for: Pan-Genome Reverse Vaccinology Approach for the Design of Multi-Epitope Vaccine Construct against Escherichia albertii
Source: Int J Mol Sci. 2021 Nov 26;22(23):12814. doi: 10.3390/ijms222312814 (PMC8657462; doi:10.3390/ijms222312814)
Supplement: Supplementary file 1 [file ijms-22-12814-s001.zip › ijms-1431045-supplementary.pdf]

Table S1. Predicted B-cell epitopes from FimD protein.

S. No.	BCPred Epitopes			FCPred			ABCPred		
	Position	Epitopes	Sores	Position	Epitopes	Scores	Position	Epitopes	Scores
**1**	649	YSVQTGYAGGGEGSNGNSGY	1	654	GYAGGGEGSNGNSG	1	718	AVLIKAPGAKDAKVEN	0.9
**2**	223	RDNTTWSYSSGANSSGSKNK	1	224	DNTTWSYSSGANSS	1	169	ARGYIPPELWDSGINA	0.9
**3**	805	NNKPLPFGAMVTSDSSQSSG	0.99	816	TSDSSQSSGIVADN	1	87	GYMATRDVTFNAGDSE	0.9
**4**	379	TAGEYRSGNAQQEKPRFFQS	0.98	321	YNSTVPPGPFTIND	1	848	GEGENAHCVADYKLPP	0.9
**5**	64	LSNFENGQELPPGTYRVDIY	0.98	760	LDTNSLADNVDLDN	1	506	DGVIQVKPKFTDYYNL	0.89
**6**	312	SIKQNGYDIYNSTVPPGPFT	0.96	192	TGNSVQNSIGGDSH	1	818	DSSQSSGIVADNGQVY	0.88
**7**	184	AGLLNYNFTGNSVQNSIGGD	0.95	66	NFENGQELPPGTYR	0.99	747	LPYATEYRENRVALDT	0.88
**8**	402	HGLPAGWTIYGGMQLADRYR	0.94	445	NATLPDDSEHEGQS	0.99	658	GGEGSNGNSGYAALNY	0.88
**9**	532	SVTQQLGRTSTLYLSGSHQT	0.93	801	TLTRNNKPLPFGAM	0.99	414	MQLADRYRAFNFGVGK	0.88
**10**	261	LTLGDGYTQGDIFDGINFRG	0.92	844	QVKWGEGENAHCVA	0.99	730	KVENQTGVRTDWRGYA	0.89
**11**	766	ADNVDLDNAVANVVPTRGAI	0.85	336	YAAGNSGDLQVTIK	0.99	802	LTRNNKPLPFGAMVTS	0.88
**12**	745	AVLPYATEYRENRVALDTNS	0.84	170	RGYIPPELWDSGIN	0.99	570	FEDINWTLSYSLTKNA	0.88
**13**	163	AFMSNHARGYIPPELWDSGI	0.83	360	VPYSSVPLLQREGH	0.99	374	TRYSITAGEYRSGNAQ	0.88
**14**	284	ASDDNMLPDSQRGFAPVIHG	0.83	381	GEYRSGNAQQEKPR	0.99	345	QVTIKEADGSTQIFTV	0.88

**15**	722	KAPGAKDAKVENQTGVRTDW	0.83	675	GGYGNANIGYSHSD	0.99	214	GLNIGAWRLRDNTTWS	0.88
**16**	88	YMATRDVTFNAGDSEQGIVP	0.81	93	DVTFNAGDSEQGIV	0.98	648	SYSVQTGYAGGGEGSN	0.87
**17**	139	TSMINEATSHLDVGQQRLNL	0.81	707	GVTLGQPLNDTAVL	0.98	635	AGLYGTLLEDNNLSYS	0.87
**18**	841	GKVQVKWGEGENAHCVADYK	0.80	482	STSGYFNFADTTYK	0.98	611	KSLWRHANASYSMSHD	0.87
**19**	573	INWTLSYSLTKNAWQKGRDR	0.77	460	RFLYNKSLNESGTN	0.97	498	SGYNIETQDGVIQVKP	0.87
**20**				567	NTAFEDINWTLSYS	0.97	77	TYRVDIYLNNGYMATR	0.85
**21**				264	GDGYTQGDIFDGIN	0.95	583	KNAWQKGRDRMLALNV	0.85
**22**				284	ASDDNMLPDSQRGF	0.94	454	HEGQSVRFLYNKSLNE	0.85
**23**				402	HGLPAGWTIYGGMQ	0.92	40	APFSYADLYFNPRFLA	0.85
**24**				564	SGSHQTYWGTNNID	0.85	309	AQVSIKQNGYDIYNST	0.85
**25**				40	APFSYADLYFNPRF	0.84	140	SMINEATSHLDVGQQR	0.85
**26**				690	IKQFYYGISGGVLA	0.83	675	GGYGNANIGYSHSDDI	0.85
**27**				722	KAPGAKDAKVENQT	0.80	478	GYRYSTSGYFNFADTT	0.85
**28**				775	VANVVPTRGAIVRA	0.79	184	AGLLNYNFTGNSVQNS	0.84
**29**				531	FSVTQQLGRTSTLY	0.78	129	LLAVDACVPLTSMINE	0.84
**30**				134	ACVPLTSMINEATS	0.72	842	KVQVKWGEGENAHCVA	0.83
**31**				512	KPKFTDYYNLAYNK	0.72	71	QELPPGTYRVDIYLNN	0.83
**32**				4	LNLSVYQRNTQCLH	0.70	619	ASYSMSHDLKGRMTNL	0.83
**33**							196	VQNSIGGDSHYAYLNL	0.83
**34**							808	PLPFGAMVTSDSSQSS	0.82
**35**							629	GRMTNLAGLYGTLLED	0.82

**36**	333	NDLYAAGNS GDLQVTI	0.82
**37**	248	TWLERDIIPLR SRLTL	0.82
**38**	114	LASMGLNTAS VPGMNL	0.82
**39**	724	PGAKDAKVE NQTGVRT	0.81
**40**	549	HQTYWGTNN IDEQLQA	0.81
**41**	537	LGRTSTLYLSG SHQTY	0.8
**42**	487	FNFADTTYKR MSGYNI	0.8
**43**	221	RLRDNTTWSY SSGANS	0.8
**44**	175	PELWDSGINA GLLNYN	0.79
**45**	856	VADYKLPPES QHQLLT	0.79
**46**	791	KARVGMKLL MTLTRNN	0.79
**47**	694	YYGISGGVLA HADGVT	0.79
**48**	404	LPAGWTIYGG MQLADR	0.79
**49**	293	SQRGFAPVIH GIARGT	0.79
**50**	229	SYSSGANSSGS KNKWQ	0.79
**51**	160	IPQAFMSNHA RGYIPP	0.79
**52**	664	GNSGYAALN YRGGYGN	0.78
**53**	32	VACVFAAQA PFSYADL	0.78
**54**	261	LTLGDGYTQG DIFDGI	0.78
**55**	832	VYLSGMPLAG KVQVKW	0.77
**56**	684	YSHSDDIKQF YYGISG	0.77
**57**	526	RGKLQFSVTQ QLGRTS	0.77
**58**	269	QGDIFDGINF RGAELA	0.77
**59**	763	NSLADNVDL DNAVAN	0.76

**60**	564	AGLNTAFEDI NWTLSY	0.76
**61**	707	GVTLGQPLN DTAVLIK	0.75
**62**	517	DYYNLAYNK RGKLQFS	0.75
**63**	102	EQGIVPCLTR AQLASM	0.75
**64**	824	GIVADNGQV YLSGMPL	0.74
**65**	95	TFNAGDSEQG IVPCLT	0.73
**66**	701	VLAHADGVT LGQPLND	0.73
**67**	446	ATLPDDSEHE GQSVRF	0.73
**68**	440	DMTQANATL PDDSEHE	0.73
**69**	431	MGALGALSV DMTQANA	0.73
**70**	353	GSTQIFTVPYS SVPLL	0.73
**71**	757	RVALDTNSLA DNVDLD	0.72
**72**	51	PRFLADDPQA VADLSN	0.72
**73**	318	YDIYNSTVPP GPFTIN	0.72
**74**	9	YQRNTQCLH NRKLRLA	0.71
**75**	302	HGIARGTAQ VSIKQNG	0.71
**76**	279	RGAELASDD NMLPDSQ	0.71
**77**	202	GDSHYAYLN LQSGLNI	0.71
**78**	737	VRTDWRGYA VLPYATE	0.71
**79**	384	RSGNAQQEK PRFFQST	0.7
**80**	555	TNNIDEQLQA GLNTAF	0.68
**81**	58	PQAVADLSNF ENGQEL	0.66
**82**	25	GFFVRLSVAC VFAAQA	0.66
**83**	393	PRFFQSTLLH GLPAGW	0.65

**84**	468	NESGTNIQLV GYRYST	0.64
**85**	360	VPYSSVPLLQ REGHTR	0.64
**86**	285	SDDNMLPDS QRGFAPV	0.62
**87**	425	FGVGKNMGA LGALSVD	0.61
**88**	1	MSYLNLSVYQ RNTQCL	0.6
**89**	146	TSHLDVGQQ RLNLTIP	0.56
**90**	18	NRKLRLAGFF VRLSVA	0.54

Table S2. Common Identified B-cells Epitopes from all three tools.

S. No	Position	Epitopes	Scores
1	163	AFMSNHARGYIPPELWDSGI	0.84
2	223	RDNTTWSYSSGANSSGSKNK	1
3	801	TLTRNNKPLPFGAM	0.99
4	844	QVKWGEGENAHCVA	0.9
5	64	LSNFENGQELPPGTYRVDIY	0.9
6	87	GYMATRDVTFNAGDSE	0.9
7	816	TSDSSQSSGIVADN	1
8	841	GKVQVKWGEGENAHCVADYK	0.8
9	184	AGLLNYNFTGNSVQNS	0.9
10	129	LLAVDACVPLTSMINE	0.8
11	318	YDIYNSTVPPGPFTIN	0.7
12	374	TRYSITAGEYRSGNAQ	0.8
13	88	YMATRDVTFNAGDSEQGIVP	0.8
14	649	YSVQTGYAGGGEGSNGNSGY	1
15	760	LDTNSLADNVDLDN	1
16	402	HGLPAGWTIYGGMQ	0.9
17	32	VACVFAAQAPFSYADL	0.7
18	284	ASDDNMLPDSQRGF	0.9
19	722	KAPGAKDAKVENQTGVRTDW	0.8
20	570	FEDINWTLSYSLTKNA	0.8
21	169	ARGYIPPELWDSGINA	0.9
22	4	LNLSVYQRNTQCLH	08
23	393	PRFFQSTLLHGLPAGW	0.9
24	64	LSNFENGQELPPGTYRVDIY	0.9
25	77	TYRVDIYLNNGYMATR	0.8
26	531	FSVTQQLGRTSTLY	0.7

Table S3. Comparative analysis of B Cell, MHC-I and MHC-II epitopes.

S. No	B-cell epitopes	MHC-I	MHC-II	Scores
1	LNLSVYQRNTQCLHNRKLRLAGFF VRLSVA	YQRNTQCLH	NRKLRLAGFFVRLSV RKLRLAGFFVRLSVA	0.6
2	TAGEYRSGNAQQEKPRFFQSTLLHG LPAGWTIYGGMQLADRYR	--	KPRFFQSTLLHGLPA EKPRFFQSTLLHGLP QEKPRFFQSTLLHGL	0.8
3	LSNFENGQELPPGTYRVDIYLNNGY MATRDVTFNAGDSE	--	YRVDIYLNNGYMATR RVDIYLNNGYMATRD	0.9

Table S4. Vaccine model Constructs.

S. No	Vaccine construct	Vaccine composition	Sequence
1	V1	FimD, HBHA adjuvant, (4-33, 64-102 and 379-421)	EAAAKMAENPNIDDLPAPLLAALGAADLALATVNDLIANLRERAEETRAETRTRVEERRARLTKFQEDLPEQFIELRDKFTTEELRKAAEGYLEAATNRYNELVERGEAALQRLRSQTAFEDASARAEGYVDQAVELTQEALGTVASQTRAVGERAAKLVGIELEAAAK*AKFVAAWTLKAAA*GGGS**LNLSVYQRNTQCLHNRKLRLAGFFVRLSVA**GGGS**LSNFENGQELPPGTYRVDIYLNNGYMATRDVTFNAGDSE**GGGSHEYGAEALERAG**TAGEYRSGNAQQEKPRFFQSTLLHGLPAGWTIYGGMQLADRYR**HEYGAEALERAG*AKFVAAWTLKAAA*GGGS
2	V2	FimD, HBHA conserved adjuvant, (4-33, 64-102 and 379-421)	EAAAKMAENSNIDDIKAPLLAALGAADLALATVNELITNLRERAEETRRSRVEESRARLTKLQEDLPEQLTELREKFTAEELRKAAEGYLEAATSELVERGEAALERLRSQQSFEEVSARAEGYVDQAVELTQEALGTVASQVEGRAAKLVGIELEAAAK*AKFVAAWTLKAAA*GGGS**LNLSVYQRNTQCLHNRKLRLAGFFVRLSVAGGGSLSNFENGQELPPGTYRVDIYLNNGYMATRDVTFNAGDSE**GGGSHEYGAEALERAG**TAGEYRSGNAQQEKPRFFQSTLLHGLPAGWTIYGGMQLADRYR**HEYGAEALERAG*AKFVAAWTLKAAA*GGGS
3	V3	FimD, Beta defensin adjuvant, (4-33, 64-102 and 379-421)	EAAAKGIINTLQKYYCRVRGGRCAVLSCLPKEEQIGKCSTRGRKCCRRKKEAAAK*WTLKAAA*GGGS**LNLSVYQRNTQCLHNRKLRLAGFFVRLSVA**GGGS**LSNFENGQELPPGTYRVDIYLNNGYMATRDVTFNAGDSE**GGGSHEYGAEALERAG**TAGEYRSGNAQQEKPRFFQSTLLHGLPAGWTIYGGMQLADRYR**HEYGAEALERAG*AKFVAAWTLKAAA*GGGS
4	V4	FimD, Ribosomal adjuvant adjuvant, (4-33, 64-	EAAAKMAKLSTDELLDAFKEMTLLELSDFVKKFEETFEVTAAAPVAVAAAGAAPAGAAVEAAEEQSEFDVILEAAGDKKIGVIKVVREIVSGLGLKEAKDLVDGAPKPLLEKVAKEAADEAKAKLEAAGATVTVKEAAAK*AKFVAAWTLKAAA*GGGS**LNLSVYQRNTQCLHNRKLRLAGFFVRLSVA**GGGS**LSNFENGQELPPGTYRVDIYLNNGYMATRDVTFNAGDSE**GGGSHEYGAEALERAG**TAGEYRSGNAQQEKPRFFQSTLLHGLPAGWTIYGGMQLADRYR**HEYGAEALERAG*AKFVAAWTLKAAA*GGGS

		102 and 379-421)	
**5**	V5	FimD, HBHA adjuvant (379-421, 64-102, and 4-33)	EAAAKMAENPNIDDLPAPLLAALGAADLALATVNDLIANLRERAEETRAETRTRVEERRARLTKFQEDLPEQFIELRDKFTTEELRKAAEGYLEAATNRYNELVERGEAALQRLRSQTAFEDASARAEGYVDQAVELTQEALGTVASQTRAVGERAAKLVGIELEAAAK*AKFVAAWTLKAAA*GGGS**TAGEYRSGNAQQEKPRFFQSTLLHGLPAGWTIYGGMQLADRYR**GGGS**LSNFENGQELPPGTYRVDIYLNNGYMATRDVTFNAGDSE**HEYGAEALERAG**LNLSVYQRNTQCLHNRKLRLAGFFVRLSVA**HEYGAEALERAG*AKFVAAWTLKAAA*GGGS
**6**	V6	FimD, HBHA conserved adjuvant (379-421, 64-102, and 4-33)	EAAAKMAENSNIDDIKAPLLAALGAADLALATVNELITNLRERAEETRRSRVEESRARLTKLQEDLPEQLTELREKFTAEELRKAAEGYLEAATSELVERGEAALERLRSQQSFEEVSARAEGYVDQAVELTQEALGTVASQVEGRAAKLVGIELEAAAK*AKFVAAWTLKAAA*GGGS**TAGEYRSGNAQQEKPRFFQSTLLHGLPAGWTIYGGMQLADRYR**GGGS**LSNFENGQELPPGTYRVDIYLNNGYMATRDVTFNAGDSE**HEYGAEALERAG**LNLSVYQRNTQCLHNRKLRLAGFFVRLSVA**HEYGAEALERAG*AKFVAAWTLKAAA*GGGS
**7**	V7	FimD, Beta defensin adjuvant (379-421, 64-102, and 4-33)	EAAAKGIINTLQKYYCRVRGGRCAVLSCLPKEEQIGKCSTRGRKCCRRKKEAAAK*AKFVAAWTLKAAA*GGGS**TAGEYRSGNAQQEKPRFFQSTLLHGLPAGWTIYGGMQLADRYR**GGGS**LSNFENGQELPPGTYRVDIYLNNGYMATRDVTFNAGDSE**HEYGAEALERAG**LNLSVYQRNTQCLHNRKLRLAGFFVRLSVA**HEYGAEALERAG*AKFVAAWTLKAAA*GGGS
**8**	V8	FimD, Ribosomal adjuvant (379-421, 64-102, and 4-33)	EAAAKMAKLSTDELLDAFKEMTLLELSDFVKKFEETFEVTAAAPVAVAAAGAAPAGAAVEAAEEQSEFDVILEAAGDKKIGVIKVVREIVSGLGLKEAKDLVDGAPKPLLEKVAKEAADEAKAKLEAAGATVTVKEAAAK*AKFVAAWTLKAAA*GGGS**TAGEYRSGNAQQEKPRFFQSTLLHGLPAGWTIYGGMQLADRYR**GGGS**LSNFENGQELPPGTYRVDIYLNNGYMATRDVTFNAGDSE**HEYGAEALERAG**LNLSVYQRNTQCLHNRKLRLAGFFVRLSVA**HEYGAEALERAG*AKFVAAWTLKAAA*GGGS
**9**	V9	FimD, HBHA adjuvant, (64-102, 4-33, and 379-421)	EAAAKMAENPNIDDLPAPLLAALGAADLALATVNDLIANLRERAEETRAETRTRVEERRARLTKFQEDLPEQFIELRDKFTTEELRKAAEGYLEAATNRYNELVERGEAALQRLRSQTAFEDASARAEGYVDQAVELTQEALGTVASQTRAVGERAAKLVGIELEAAAK*AKFVAAWTLKAAA*GGGS**LSNFENGQELPPGTYRVDIYLNNGYMATRDVTFNAGDSE**GGGS**LNLSVYQRNTQCLHNRKLRLAGFFVRLSVA**HEYGAEALERAG**TAGEYRSGNAQQEKPRFFQSTLLHGLPAGWTIYGGMQLADRYR**HEYGAEALERAG*AKFVAAWTLKAAA*GGGS
**10**	V10	FimD, HBHA conserved	EAAAKMAENSNIDDIKAPLLAALGAADLALATVNELITNLRERAEETRRSRVEESRARLTKLQEDLPEQLTELREKFTAEELRKAAEGYLEAATSELVERGEAALERLRSQQSFEEVSARAEGYVDQAVELTQEALGTVASQVEGRAAKLVGIELEAAAK*AKFVAAWTLKAAA*GGGS**LSNFENGQELPPGTYRVDIYLNNG**

		adjuvant, (64-102, 4-33, and 379-421)	**YMATRDVTFNAGDSE**GGGS**LNLSVYQRNTQCLHNRKLRLAGFFVRLSVA**HEYGAEALERAG**TAGEYRSGNAQQEKPRFFQSTLLHGLPAGWTIYGGMQLADRYR**HEYGAEALERAG*AKFVAAWTLKAAA*GGGS
**11**	V11	FimD, Beta defensin adjuvant (64-102, 4-33, and 379-421)	EAAAKGIINTLQKYYCRVRGGRCAVLSCLPKEEQIGKCSTRGRKCCRRKKEAAAK*AKFVAAWTLKAAA*GGGS**LSNFENGQELPPGTYRVDIYLNNGYMATRDVTFNAGDSE**GGGS**LNLSVYQRNTQCLHNRKLRLAGFFVRLSVA**HEYGAEALERAG**TAGEYRSGNAQQEKPRFFQSTLLHGLPAGWTIYGGMQLADRYR**HEYGAEALERAG*AKFVAAWTLKAAA*GGGS
**12**	V12	FimD, Ribosomal adjuvant (64-102, 4-33, and 379-421)	EAAAKMAKLSTDELLDAFKEMTLLELSDFVKKFEETFEVTAAAPVAVAAAGAAPAGAAVEAAEEQSEFDVILEAAGDKKIGVIKVVREIVSGLGLKEAKDLVDGAPKPLLEKVAKEAADEAKAKLEAAGATVTVKEAAAK*AKFVAAWTLKAAA*GGGS**LSNFENGQELPPGTYRVDIYLNNGYMATRDVTFNAGDSE**GGGS**LNLSVYQRNTQCLHNRKLRLAGFFVRLSVA**HEYGAEALERAG**TAGEYRSGNAQQEKPRFFQSTLLHGLPAGWTIYGGMQLADRYR**HEYGAEALERAG*AKFVAAWTLKAAA*GGGS

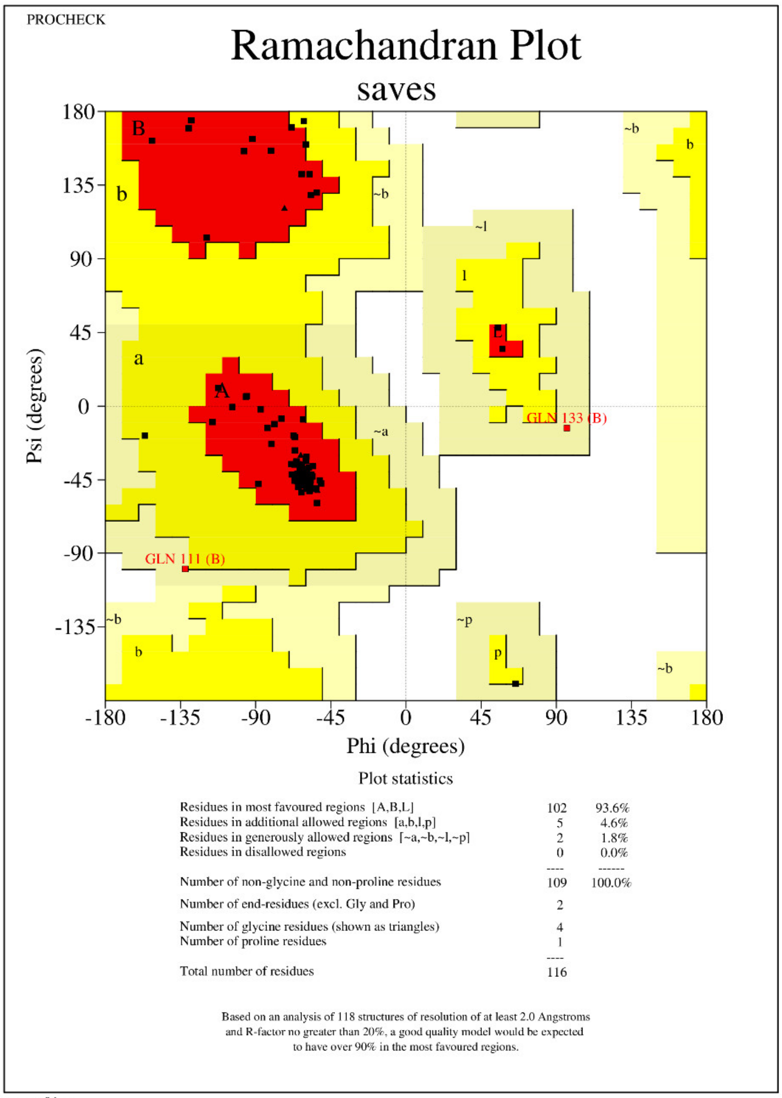

saves_01.ps

Figure S1A. Ramachandran plot of V6.

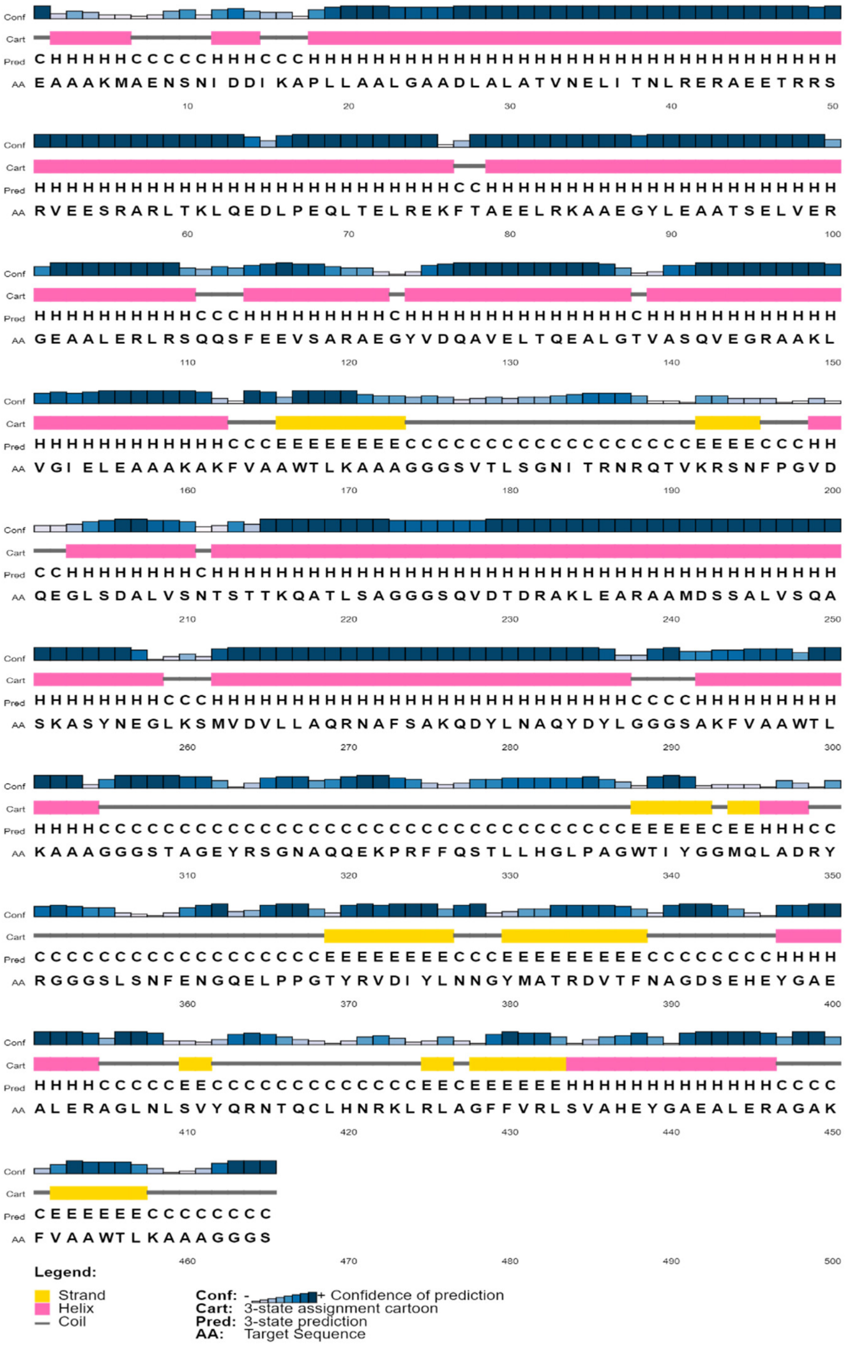

Figure S1B. Secondary structure of V6 construct.

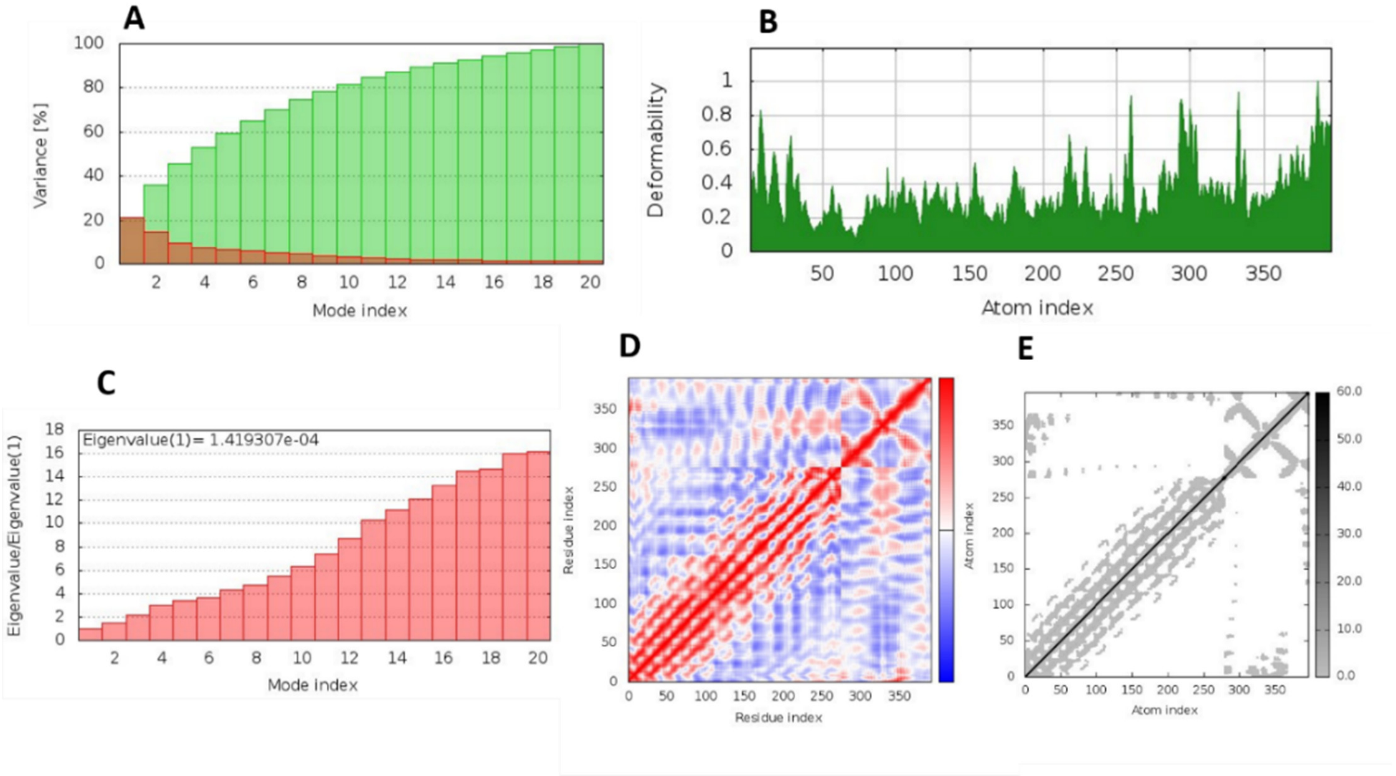

Figure S2. Secondary structure of V6 construct.
